# #TheSkinnyaboutSkin: how good are we at assessing all skin colours?

**DOI:** 10.29045/14784726.2022.03.6.4.60

**Published:** 2022-03-01

**Authors:** Juliet Harrison

**Affiliations:** University of East Anglia

Given the COVID-19 pandemic, you may be aware that health outcomes in the United Kingdom may be poorer for those who do not have white skin (Raleigh & Holmes, 2021). Reasons such as socio-economic factors have been proposed (Raleigh & Holmes, 2021), but another possibility I suggest is an unconscious inequality in patient clinical assessment. Take a moment and consider: how good are we at assessing all skin colours? Part of a paramedic’s role is to rapidly recognise a deteriorating patient, but are we held back because healthcare training has historically related to assessing patients with white skin? Think about the widespread white-based terminology we use – for example ‘pink & perfused’, or signs of ‘reddening’ in inflammation – which embeds white skin- orientated assessment. Recognising cyanosis in all skin colours is a skill as it is different, as is assessing rashes, contusions . . . and so the list continues. Textbooks may not be helpful, with pictures of white patient presentations as the norm, except if it is a condition prevalent in certain ethnicities. And this leads me to query that if we lack training, do we then rely on kit to tell us how unwell a patient is? A study by Sjoding et al. (2020) found black patients were nearly three times more likely to have an undetected hypoxaemia. This was found by comparing pulse oximetry with simultaneous arterial blood gas readings and finding discrepancies – for example, finding an Sp02 of 92–96% with a Pa02 of below 88%. If clinicians are not detecting signs of deterioration in all skin colours successfully, then there is an issue that we need to be addressing nationally around patient safety. This is the basis of my PhD research; see #TheSkinnyaboutSkin on Twitter. And, if you are interested in resources to improve knowledge, try ‘Mind the Gap’ at https://www.blackandbrownskin.co.uk.
